# Dehydrated citrus pulp in rabbit feeding

**DOI:** 10.1007/s11250-023-03696-z

**Published:** 2023-10-04

**Authors:** Juliana A. Rubio Varela, Mayra Diaz-Vargas, Carlos Felipe Duque-Ramírez, Lina Maria Peñuela Sierra

**Affiliations:** 1 Facultad de Ciencias Agropecuarias, Universidad de Ciencias Aplicadas Y Ambientales U.D.C.A, Bogotá, Colombia; 2 Universidad del Tolima, Universidad del Tolima, Facultad de Medicina Veterinaria Y Zootecnia, Grupo de Investigación en Sistemas Agroforestales Pecuarios, Ibagué, Colombia

**Keywords:** By-product, Performance, Quality, Substitution

## Abstract

The objective of this study was to evaluate the effect of dehydrated orange by-product on the productive parameters, carcass yield, and meat quality of rabbits in the fattening stage. Four diets with increasing levels of orange by-products (0%, 10%, 20%, and 30%) were evaluated, each with six replicates of two 32-day-old crossbred rabbits that were randomly allocated. The experimental period was from 32 to 70 days of age of the rabbits. A higher weight gain (*p* < 0.05) was achieved for observed with 20% substitution, along with a better feed conversion, without any effects on carcass yield and meat quality parameters. The treatment with 30% citrus pulp showed no differences (*p* > 0.05) in the parameters evaluated. Based on our results, dehydrated orange by-product can replace up to 30% of the commercial feed concentrate without affecting performance, yield, and carcass quality in rabbits feeding since 32 to 72 days of age.

## Introduction

Rabbit farming is currently receiving wide attention because of the short cycle, high prolificacy, and easy management of rabbits. In addition, rabbit meat is increasingly being accepted as an option of high nutritional value; the production of rabbit meat in Colombia is between 5000 and 19,000 tons per year with a per capita consumption of 0.24% (Laverde 2021). The particularities of the gastrointestinal tract of rabbits allow the use of fibrous sources in the diet and the application of non-conventional feeding systems. According to Trocino et al. (2013), total dietary fiber is the major fraction of commercial diets for rabbits (35–50% as-fed). De Maria et al. (2013) reported the importance of the caecum in this species since it increases fiber digestibility and short-chain fatty acid absorption. The gastrointestinal characteristics of rabbits enable the use of alternatives to reduce feed costs, in this case, focusing on agri-food by-products that can be an important source in animal feed, either as an energy or protein component. Likewise, their properties provide benefits to animal products. On the other hand, Ferreira et al. (2019) found evidences the important role of fibrous fractions as well as non-fibrous carbohydrates on the fermentative behavior of the ingredients with important impact on the total amount of gas produced, and on the digestibility, and degradability of the ingredients in diets for rabbits.

Feeding monogastric species is one of the major production constraints because it can account for 70% of the total costs (Araujo et al. 2008). Recently, the use of raw materials such as corn, alfalfa hay, and soybean meal, which are some of the most used ones in rabbit feed, has increased considerably. This has led to an increase in the cost of feed and in the fluctuation of prices at certain times of the year. The growth of agribusinesses and the increase in the production of by-products from these industries encourage the use of agricultural by-products in animal feed (Gomes et al., 2021). Agricultural activity generates 72 million tons of waste per year, including coffee, palm oil, corn, banana, and citrus by-products, and in many cases, these products are discarded (Bedoya et al., 2021). Faostat (2017) reported that the citrus production is a major sector worldwide, covering a stable surface of almost 10 million hectares and output of roughly 150 million tons. From the different citrus fruits, orange (50% of total production) is the most important, followed by tangerines (30% of total), lemons (12%), and pomelo (8%). China and Brazil are the main producers, but countries in the Mediterranean area are also relevant. Hassan et al. (2021) reported a considerable interest in the use of additives or natural ingredients to improve yields, highlighting citrus residues as potential antioxidant resources since they contain significant amounts of ascorbic acid, catechol, dimethoxy phenol, cyclohexane, acetic acid, stigmasterol, sitosterol, and vitamin E.

The aim of the current study was to evaluate the effect of dehydrated orange by-product on the productive parameters, carcass yield, and meat quality of rabbits in the fattening stage.

## Materials and methods

This study was conducted in the rabbit breeding area of the academic unit “The Remanso” of the University of Applied and Environmental Sciences U.D.C.A., Bogotá D.C., Colombia. Average elevation is 2630 m above sea level, with an average annual temperature of 20 °C ± 2 °C, an average annual precipitation of 797 mm, and a relative humidity that fluctuates between 77 and 83% (IDEAM, 2020). The study was approved by the Ethics Committee for Animal Research — CEEA/UDCA; 48 mongrel rabbits 32 days of age, with an average weight of 871.54 ± 88.32 g, were used. Each treatment consisted of 12 animals (6 males and 6 females), which were randomly distributed in four treatments, 0% (control), 10, 20, and 30% substitution of a commercial concentrate of dehydrated citrus pulp, composed of peel, membranes, and seeds of the Tangelo and Valencia varieties (Table 1). These materials were obtained from orange juice stands, air-dried in a covered place, and stirred three times a day for 6 days until a moisture level of 13% was reached. The bromatological composition of the citrus pulp used was 7.4% crude protein, 14.87% crude fiber, 3.8% ethereal extract, 5.1% mineral matter, 68.82% nitrogen-free extract, 16.8% neutral detergent fiber, 25.81% acid detergent fiber, 94.9% organic matter, 0.39% total polyphenols, and 3783 kcal/kg of digestible energy; these data were obtained from a digestibility test previously performed. The animals were housed in wire cages with an automatic drinking trough and a manual feeder until 70 days of life, and fed twice a day, in the morning and in the afternoon. The amount of feed supplied was adjusted according to live weight based on the NRC, 1977 indications, and water was provided ad libitum; evaluation was performed in two feeding phases (32 to 50 days and 50 to 70 days). Rabbits and experimental diets were weighed at 32, 50, and 70 days old to evaluate performance, which was measured as the weight gain, and feed conversion rate.

**Table 1:** Chemical composition of experimental diets

Item	Levels of citrus pulp inclusion (g kg^−1^)			
	0	10	20	30
Dry matter (%)	88.27	88.28	89.41	89.56
Crude protein (%)	20.05	17.85	17.81	16.77
Crude fiber (%)	13.48	13.58	14.25	16.22
Ether extract (%)	5.02	5.07	5.71	5.76
Ash (%)	12.30	11.61	10.50	10.23
Acid detergent fiber (%)	18.10	17.39	17.32	17.15
Neutral detergent fiber (%)	44.11	41.39	41.35	41.26
Gross energy (kcal kg^−1^)	4276	4320	4331	4356

At 50 and 70 days of life, five animals per treatment were slaughtered using CO_2_ desensitization and subsequent bleeding, in accordance with the regulations for animal slaughter established by the European Commission. At the end of the experiment (70 days), the animals were subjected to a fasting period (8 h), followed by sacrificing via CO_2_, skin removal, and evisceration for carcass yield (without limbs and head) and its relation to live weight and the weight of commercial cuts (loin, thighs, shoulder, and rib) as well as its relation to carcass weight. The loin muscle (longissimus lumborum) was then collected and refrigerated for 24 h, after which pH, water-holding capacity—WHC, and cooking loss were determined. For pH measurement, a Testo® contact potentiometer (model 205) was used, inserted directly, and water-holding capacity was measured using the centrifugation method proposed by Nakamura and Katok (1985). Analysis of water loss by cooking was carried out according to Honikel (1998).

The obtained results were analyzed using the SAS 9.4 statistical software; each parameter was subjected to analysis of variance (ANOVA), considering Bartl\ett’s test to verify homogeneity of variances, and the Jarque–Bera normality test was used to verify compliance with normal distribution; whenever a difference was found, the degrees of freedom were decomposed into polynomials and analyzed by regression for the different relationships—linear or quadratic (*p* < 0.05). For the variables that showed quadratic responses, the point of inflection was calculated as the best relationship. To compare the results, the data were subjected to Dunnett’s test at 5% probability, to compare each of treatments with control.





where *Yij* is the observation of the dependent variable in the experimental unit *j* submitted to level *i* of the by-product; *i* is the level of citrus pulp (0%, 10%, 20%, and 30%); *b*0 is the constant; *b*1 and *b*2 are, respectively, linear and quadratic regression coefficients of the dependent variable as a function of by-product levels; and *eij* is the random error associated with each observation *Yij*.

## Results

### Performance

When evaluating the productive parameters (Table 2), by comparing the diets with citrus pulp substitution (10%, 20%, and 30%) with the control diet, the 20% diet presented a difference in weight gain and feed conversion (*p* < 0.05) in both evaluation periods, presenting higher weight gain and better feed conversion.

**Table 2:** Performance (mean ± standard error) of 32- to 50- and 50- to 70-day-old rabbits fed diets with increasing levels of dehydrated citrus pulp

	Citrus pulp substitution levels					
	0%	10%	20%	30%	*R*^2^	*R*
32–50 days						
Weight gain (g)	801.11 ± 12.48	830.33 ± 23.42	1,063.86 ± 26.36	1,007.67 ± 26.72*	0.88	*L* = 0.01^1^
Feed conversion	2.89 ± 0.13	2.84 ± 0.17	2.19 ± 0.13	2.34 ± 0.18*	0.98	*L* = 0.01^2^
50–70 days						
Weight gain (g)	453.11 ± 7.23	425.44 ± 16.45	343.43 ± 9.99	462.00 ± 21.29*	0.80	*Q* = 0.02^3^
Feed conversion	4.31 ± 0.17	4.71 ± 0.34	5.73 ± 0.30	4.28 ± 0.29*	0.85	*Q* = 0.02^4^

*Significant by Dunnett’s test at 5%; *R*, regression; *CV*, coefficient of variation, *L*, linear; *Q*, quadratic

^1^*y* = 9.3045*x* + 781.65; *R*^2^ = 0.8792, ^2^*y* =  − 0.0254*x* + 2.891; *R*^2^ = 0.9817, ^3^*y* = 0.435 × 2 − 12.7*x* + 462.95; *R*^2^ = 0.7974; 14.59, ^4^*y* =  − 0.0055 × 2 + 0.1701*x* + 4.2065; *R*^2^ = 0.8502; 15.46

In the period from 32 to 50 days, an increasing linear effect (*p* < 0.05) of weight gain was observed, along with a decreasing feed conversion. For the period from 50 to 70 days, a quadratic effect (*p* < 0.05) of weight gain was observed with the lowest value presented in the estimated percentage of 11.87% of citrus pulp substitution and with feed conversion, with the highest conversion observed in the estimated percentage of 16.43% of citrus pulp substitution.

### Carcass yield

No effect of citrus pulp substitution levels was observed for the variables of carcass yield and cuts (Table 3). However, when comparing the substitution levels with the control treatment, higher percentages of carcass, loin, thigh, rib, and shoulder were observed for the animals fed the 30% substitution diet.

**Table 3:** Carcass yield and cuts (%) (mean ± standard error) of rabbits fed diets with increasing levels of dehydrated citrus pulp and slaughtered at 70 days of age

	Citrus pulp substitution levels				*R*^2^	*R*
	0%	10%	20%	30%		
Carcass	50.25 ± 2.11	56.19 ± 0.89*	54.92 ± 1.02*	51.14 ± 4.65*	0.00	*p* > 0.05
Thighs	36.17 ± 2.01	37.14 ± 1.43	35.65 ± 1.46	38.04 ± 0.98*	0.06	*p* > 0.05
Shoulders	12.90 ± 0.55	13.20 ± 0.33	12.34 ± 0.56	13.55 ± 0.69*	0.05	*p* > 0.05
Rib	17.21 ± 1.22	18.04 ± 1.90	20.24 ± 5.12*	18.66 ± 0.82*	0.02	*p* > 0.05
Loin	23.60 ± 0.51	25.20 ± 0.33	25.71 ± 1.00*	25.15 ± 0.94*	0.11	*p* > 0.05

*Significant by Dunnett’s test at 5%; *R*, regression; *CV*, coefficient of variation; *ns*, nonsignificant

### Meat quality

Meat quality was based on water-holding capacity (WHC), cooking loss (CWL), and pH of the loin muscle (longissimus lumborum). When comparing the substitution percentages with the control diet in terms of these parameters (Table 4), there was no significant difference (*p* > 0.05).

**Table 4:** Loin meat quality parameters (mean ± standard error) of rabbits fed diets with increasing levels of dehydrated citrus pulp and slaughtered at 70 days of age

	Citrus pulp substitution levels				*R*^2^	*R*
	0%	10%	20%	30%		
CWL	24.18 ± 1.69	23.65 ± 1.42	25.50 ± 0.51	21.47 ± 0.33	0.06	*p* > 0.05
WHC	76.61 ± 1.23	77.49 ± 1.00	78.27 ± 1.08	77.72 ± 1.29	0.04	*p* > 0.05
pH	5.73 ± 0.04	5.78 ± 0.02	5.88 ± 0.07	5.80 ± 0.04	0.08	*p* > 0.05

*CWL*, cooking weight loss; *WHC*, water-holding capacity;* R*, regression; *CV*, coefficient of variation; *ns*, nonsignificant

## Discussion

### Performance

The average daily weight gain in farmed rabbits ranges from 30 to 40 g, depending on genetic factors, and the feed conversion ratio is 3.35 to 3.45, increasing with age (Lebas et al. 1996). In this study, the change in the feed conversion ratio from the 32–50-day period to the 50–70-day period was characterized by a lower weight gain and a substantial increase in feed conversion. Similar results have been observed in a study conducted with different forage resources in the feeding of rabbits by Castaño and Cardona (2015), obtaining values of 30–47 g/day/rabbit; these authors therefore stated that weight gain decreases linearly with age. In 2018, Lu et al. studied the optimal fraction (0, 7, 14, and 21%) of citrus pulp in daily feed of rabbits and investigated how dietary citrus pulp could influence growth performance, and the authors found that citrus pulp could be used as an available feed resource for rabbits. Up to 21% dried citrus pulp in diet of rabbits had no adverse effects on growth performance.

### Carcass yield

Similar to the findings of our study, Coloni et al. (2012) reported no differences in carcass yield with different inclusion levels of citrus pulp in diets for guinea pigs and rabbits. Lu et al. (2018) found that the weights of slaughtered rabbits were close to average weights, so live weight and hot carcass in the 21% citrus pulp groups were higher than those in the control, and demonstrated the same weight trend at the end of the experiment.

Sánchez-Bustos et al. (2021), when evaluating the effects of non-conventional forage (*Erythrina edulis* and *Alocasia macrorrhiza*) substituting commercial concentrate, found equivalent results; however, the authors reported a carcass yield of 49.54%, which is lower than that in the present study. This difference can be explained by the higher energy and protein levels of citrus pulp. According to the chemical composition of the dehydrated orange by-product, when comparing the gross energy value of the citrus pulp used in this study (4308 kcal/kg) with the gross energy of corn (3861 kcal/kg); as pointed out previously (Rostagno et al. 2017), the gross energy value of citrus pulp is 11.57% higher than that of corn. Also, when comparing the protein value, citrus pulp has 6.93% more crude protein than corn and an ether extract value equivalent to that reported for corn (3.81%), indicating that it is an excellent source of energy.

A behavior like this study was observed by Torres et al. (2018) when evaluating the use of *Moringa oleifera* in rabbit feeding and achieving higher yields of hindquarter, thighs, and loin when using 19.4%. It is important to highlight that yields can vary among different breeds, based on feeding practices and age (Lebas et al. 1996). Hassan et al. (2021) found beneficial effects of citrus pulp on the carcass, reflected in the reduction of abdominal, renal, and dorsal fat in rabbit carcasses, which is attributed to the contribution of ascorbic acid from citrus pulp (200 mg/kg), preventing lipoperoxidation.

### Meat quality

The pH, CWL, and WRC values were within the ranges reported as optimum for rabbit carcasses. The positive effects on meat quality are for mainly regarding the oxidative stability of meat, avoiding the use of synthetic additives (Serra et al., 2021). It is important to highlight that the improvement in oxidative stability is related to the decrease in carcass protein alteration, thus ensuring a greater amount of available amino acids (Valenzuela and Pérez 2016). Water-holding capacity influences meat color, juiciness, and texture (Sierra 2006) and is a critical parameter in profitability. Leal and Jiménez (2015) indicate that an increase in water losses in meat (shrinkage) decreases the value of the end product by reducing the quality of the product. In the case of pH, it is a crucial characteristic of meat quality since it affects the protein property. The optimal pH range is between 5.65 and 5.9, and the values obtained in this study (5.73 to 5.88) are within this range (Aparicio et al. 2021; Sierra 2006).

In conclusion, dehydrated orange by-product can be used in rabbit diets in the fattening phase at an inclusion level of up to 30%, resulting in better weight gain and feed conversion and without altering carcass yield and carcass cuts and quality. This approach can therefore be used in the fattening of rabbits from 32 to 70 days of life.

## Data Availability

Not applicable.
